# A Review on the Antimicrobial Activity of Schiff Bases: Data Collection and Recent Studies

**DOI:** 10.3390/antibiotics11020191

**Published:** 2022-02-01

**Authors:** Jessica Ceramella, Domenico Iacopetta, Alessia Catalano, Francesca Cirillo, Rosamaria Lappano, Maria Stefania Sinicropi

**Affiliations:** 1 Department of Pharmacy, Health and Nutritional Sciences, University of Calabria, 87036 Arcavacata di Rende, Italy; jessica.ceramella@unical.it (J.C.); domenico.iacopetta@unical.it (D.I.); francesca.cirillo@unical.it (F.C.); rosamaria.lappano@unical.it (R.L.); s.sinicropi@unical.it (M.S.S.); 2 Department of Pharmacy-Drug Sciences, University of Bari “Aldo Moro”, 70126 Bari, Italy

**Keywords:** Schiff bases, antibacterials, imine, nanoparticles, chitosan, antimicrobials, chitosan-based Schiff bases

## Abstract

Schiff bases (SBs) have extensive applications in different fields such as analytical, inorganic and organic chemistry. They are used as dyes, catalysts, polymer stabilizers, luminescence chemosensors, catalyzers in the fixation of CO_2_ biolubricant additives and have been suggested for solar energy applications as well. Further, a wide range of pharmacological and biological applications, such as antimalarial, antiproliferative, analgesic, anti-inflammatory, antiviral, antipyretic, antibacterial and antifungal uses, emphasize the need for SB synthesis. Several SBs conjugated with chitosan have been studied in order to enhance the antibacterial activity of chitosan. Moreover, the use of the nanoparticles of SBs may improve their antimicrobial effects. Herein, we provide an analytical overview of the antibacterial and antifungal properties of SBs and chitosan-based SBs as well as SBs-functionalized nanoparticles. The most relevant and recent literature was reviewed for this purpose.

## 1. Introduction

**Figure 1:**
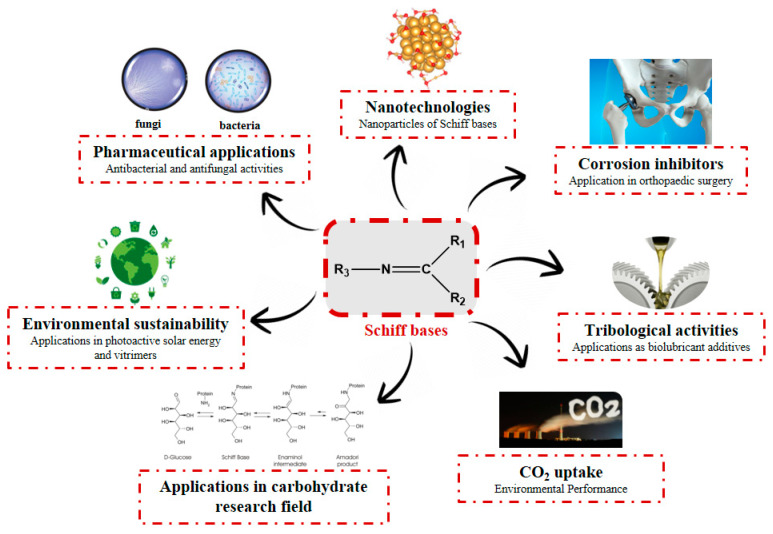
Main properties and uses of Schiff bases.

Schiff bases (SBs) are organic compounds characterized by an imine or azomethine group (>C=N–) that are widely used as pigments and dyes, catalysts, polymer stabilizers [1], luminescence chemosensors [2] and intermediates in organic synthesis [3]. SBs can also be used as corrosion inhibitors for different metal–electrolyte systems, since they adsorb and form a corrosion-mitigating surface film through their electron-rich centers, including the imine moiety. In fact, this moiety can offer strong bonding with metallic ions because of its π-acceptor properties [4,5,6]. Moreover, several studies addressed the tribological activities of SBs and their role as biolubricant additives [7,8,9,10]. Additionally, the use of SBs as catalysts in fixation of CO_2_ to mitigate its accumulation in the atmosphere has been widely described [11]. SBs have also been studied in the carbohydrate research field in relation to Amadori products [12] and in material chemistry for applications in photoactive solar energy [13,14] and vitrimers [15]. Moreover, chemical reactions involving SBs may be found in sensors used to analyze illicit drugs and determine analytes in seized samples [16]. The exceptional popularity of SBs in organic chemistry can be attributed to their simple synthesis techniques, which use cheap materials. In fact, different synthetic routes continue to be described in the literature [17,18,19]. In biological chemistry, SBs have demonstrated a broad range of biological activities [20], including antimalarial, antiproliferative [21], analgesic, anti-inflammatory [22], antiviral, antipyretic, antifungal [23] and antibacterial [24] properties (Figure 1). The imine or azomethine group (>C=N–) seems to be critical for their biological activities.

Recently, evidence on anticancer effects elicited by SBs has been provided [25,26]. It is worth noting that, in most cases, higher activity has been reported for SBs–metals complexes, rather than SBs alone [27,28]. Indeed, the presence of unpaired electrons on the nitrogen atom of the azomethine moiety gives strong chelating ability to SBs. SBs are now considered interesting ligands for coordination chemistry, thanks to their chelating ability along with, the convenience of separation and flexibility on the C=N group [29]. In recent years, the phenomenon of antibiotic resistance in hospitals, communities and environment has increasingly grown. It is known that the overuse of antibiotics has led to an increase in bacterial resistance [30] and, in turn, to decreasing efficiency of the rare available antibiotics [31,32]. It is noteworthy that, also in this case, the complexation of SBs with metals generally leads to enhanced antibacterial and antifungal effects in comparison with free SBs [33,34,35]. Recently, a novel and green cellulose-based antibacterial complex of an SB with copper was fabricated. Its antibacterial activity against *Escherichia coli* and *Staphylococcus aureus* increased by 472% and 823%, respectively, in comparison to the SB ligand [36]. However, SBs complexes are not totally devoid of disadvantages. For instance, when using SBs as homogeneous catalysts there can be reduction in the activation level in the process, demanding the recovery and recycling of catalyst and metal contaminations in products [37]. Moreover, the keto–enolic tautomeric isomerism that may occur at the double bond C=N, in the presence of *ortho*-hydroxy groups, may lead to different activities for the two tautomers [38]. Considerable interest has also been recently devoted to chitosan-based Schiff bases (CBSs) for their application in various fields such as biology, catalysis, sensors and water treatment [39]. Chitosan is a polysaccharide of natural origin, obtained from marine crustaceans, mollusks, insects and fungi. Of note, chitosan has shown antitumor, antiulcer, immunostimulatory, antidiabetic, antioxidant and antibacterial activities [40]. Recently, chitosan and its derivatives have also received attention as adsorbents for heavy metal ions and their removal from wastewater [41,42,43]. Nanoparticles represent an excellent platform for a broad range of biological and biomedical applications. Schiff base-functionalized nanoparticles have often been used for the determination of heavy metal ions [44]. Recently, the synthesis of core–shell (Fe_3_O_4_@SiO_2_) magnetic nanoparticles functionalized using an SB ligand for uranium extraction has also been reported [45]. Currently, nanoparticles may represent a promising approach for fighting antimicrobial resistance [46]. In this scenario, and given the need to identify new therapeutics in the antimicrobial field, this narrative review aims at highlighting the recent studies describing the molecular mechanisms involved in the antibacterial and antifungal effects elicited by SBs and CBSs as well as SB- and CBS-based nanoparticles.

## 2. Schiff Bases as Antimicrobial Agents

The antibacterial and antifungal activity of some SBs has been reported. The minimal inhibitory concentration (MIC) and/or inhibition zone diameter (IZD) are given.

### 2.1. Schiff Bases with Antibacterial Activity

A recent work by Hassan et al. (2019) [47] on SBs derived from 5-aminopyrazoles, namely 5-(benzylideneamino)-3-(4-methoxyphenylamino)-*N*-phenyl-1*H*-pyrazole-4-carboxamides, showed their antimicrobial activity against multidrug-resistant bacteria (MDRB). In particular, compounds **1**–**3** (Table 1) were more active than ciprofloxacin against Gram-positive *Staphylococcus epidermidis* (MIC = 7.81 µg/mL versus 15.62 µg/mL of ciprofloxacin), while compound **4** had the same activity of the reference against *Enterococcus faecalis* (MIC = 7.81 µg/mL), which is a Gram-positive organism responsible for serious infections in humans [48]. Interestingly, compounds **2** and **3** were also active against Gram-negative *Acinetobacter baumanni*, showing the same MIC values of ciprofloxacin (MIC = 15.62 µg/mL). Moreover, in silico ADMET studies were carried out. Enzymatic assays and molecular docking revealed that compound **3** exerted a strong inhibitory activity against *S. aureus* DNA gyrase and dihydrofolate reductase kinases.

Recently, Gümüş et al. (2020) [49] described two series of SB derivatives with anthracene- and pyrene-based units, which were synthesized and evaluated for their antibacterial activity against Gram-negative *Bacillus cereus, E. coli* and *Pseudomonas aeruginosa*. These experiments were performed by using the disk diffusion method taking tetracycline (30 μg) and streptomycin (10 μg) discs as positive controls. DNA binding activities were also tested by agarose gel electrophoresis, in which DNA molecules behave in accordance with their mass, charge and shape. As a result of binding the compounds to calf thymus DNA (CT-DNA), free DNA moves faster on the gel as it is smaller in respect to bound DNA. Of note, compounds **5** and **6** (*E*)-2-((anthracen-1-ylimino)methyl)quinolin-8-ol and (*E*)-2-((pyren-1-ylimino)methyl)pyridin-3-ol, respectively, which were found to be stably bound to CT-DNA, showed antibacterial activity against *Bacillus cereus* and *E. coli*.

Erturk et al. (2020) [50] synthesized two SBs, namely 4-(((8-hydroxyquinolin-2-yl)methylene)amino)-1,5-dimethyl-2-phenyl-1,2-dihydro-3*H*-pyrazol-3-one and 4-(((10-chloroanthracen-9-yl)methylene)amino)-1,5-dimethyl-2-phenyl-1,2-dihydro-3*H*-pyrazol-3-one (**7** and **8**, respectively), that were studied for their antimicrobial and antioxidant properties. Both compounds proved high antibacterial activity against Gram-positive bacteria *Micrococcus luteus* and *S. aureus* (MIC = 25 μg/mL and 12.5 μg/mL, respectively, versus MIC = 100 μg/mL and 12.5 μg/mL of ampicillin). In addition, compound **8** exhibited high antifungal potential against fungi *Aspergillus niger* (MIC = 12.5 μg/mL versus MIC = 12.5 μg/mL of nystatin). They also showed antiproliferative activity against the MCF-7 human breast cancer cell line (IC_50_ < 0.1 mM and IC_50_ = 0.14 mM for **7** and **8**, respectively).

In the study of Mishra et al. (2020) [51] the synthesis and antibacterial evaluation of two SB ligands comprising benzothiazole derivatives, namely (*N*,*N′*,*E*,*N*,*N′E*)-*N*,*N*′-(1,3-phenylenebis(methanylylidene))bis(5-nitrobenzo[d]thiazoL2-amine (**9**) and (*N*,*N′*,*E*,*N*,*N′E*)-*N*,*N*′-(1,3-phenylene-bis(methanylylidene))bis(5-methylthiazo-L2-amine (**10**) and their lanthanide (III) complexes were described. Ligands showed antibacterial activity against *S. aureus* causing skin infection and food poisoning and pimple-causing *Propionic bacteria acnes*. Of note, the complexes were more active than SBs alone.

Yusuf et al. (2020) [52] reported the synthesis and antimicrobial evaluation of three SBs, (*E*)-1-(2-nitrophenyl)-*N*-(*o*-tolyl)methanimine (**11**), (*E*)-2-isopropyl-*N*-(2-nitrobenzylidene)aniline (**12**) and (*E*)-2-((2-nitrobenzylidene)amino)phenol (**13**). Compounds **11**–**13** were tested against *E. coli*, *Salmonella typhimurium* and *P. aeruginosa* as shown in Table 1. Then, compounds **11**–**13** were docked into the active site pocket as observed experimentally of crystalized structures from *E. coli*, *S. typhimurium*, *P. aeruginosa* and *S. aureus.* Compound **12** showed the highest inhibitory activity against the Gram-negative bacteria *S. thyphymurium* and *P. aeruginosa* (MIC = 15.625 µg/mL and 7.81 µg/mL, respectively) compared to the reference drug (chloramphenicol: MIC = 31.25 µg/mL and 62.50 µg/mL, respectively).

Interesting findings on *P. aeruginosa* were also reported by Chemchem et al. (2020) [53] for twelve isatin SBs obtained by green synthesis. The compounds were studied against standard and clinical bacterial strains by the agar-well diffusion method. Compound **14** and **15** revealed the lowest MIC (625 μg/mL) against standard *S. aureus* 25923 and *Klebsiella pneumoniae* 700,603, respectively, while the reference compound colistin was inactive. When tested against *P. aeruginosa* clinical bacterial strains, compounds **16** and **14** were the most active, showing an MIC value of 78 μg/mL with a large IZD (24 mm and 15 mm, respectively), compared to the antibiotic fosfomycin (IZD = 16 mm). QSAR studies showed that increased hydrophobic character associated with decreased dipole moment led to higher antibacterial activity against the standard *K. pneumoniae* strain.

Warad et al. (2020) [54] reported the synthesis of three SBs and their antibacterial evaluation by means of the disk diffusion test. The compounds showed low activity with respect to gentamicin. The most interesting compound was 2-(piperazin-1-yl)-*N*-(thiophen-2-ylmethylene)-ethanamine (**17**). The most sensitive isolates to this compound were Gram-positive *S. aureus* and methicillin-resistant *S. aureus* (MRSA), as shown in Table 1, compared to gentamicin (IZD = 25 and 24 mm, respectively) and *P. aeruginosa* (gentamicin, IZD = 16 mm).

Bayeh at al. (2020) [55] described the synthesis of three SBs and their antimicrobial activities compared to ciprofloxacin and chloramphenicol, as references. Among the SBs, **18** showed higher activity (IZD = 32 mm) than the references against Gram-positive *S. epidermidis* (IZD = 24 and 26.7 mm for ciprofloxacin and chloramphenicol, respectively) and against Gram-negative *P. aeruginosa* (IZD = 21.3 and 27.3 mm for ciprofloxacin and chloramphenicol, respectively), whereas compound **19** was more active against *S. aureus* (IZD = 32.5 mm) in respect to both ciprofloxacin and chloramphenicol (IZD = 24 and 26.3 mm, respectively).

Anwar et al. (2020) [56] described the synthesis of SBs of amikacin and their screening for in vitro antibacterial activity by the well diffusion method against *Bacillus megaterium, Bacillus subtilis, Stenotrophomonas maltophilia, S. aureus, M. luteus, Serratia marcescens* and *E. coli.* The authors concluded that derivatives of amikacin with aromatic rings were more active antibacterials than those with an aliphatic side chain. The most interesting compounds were **20** and **21**, which showed higher activity against *S. aureus* (IZD = 37 and 321 mm, respectively) than amikacin (IZD = 29 mm) and *E. coli* (IZD = 37 and 38 mm versus amikacin IZD = 25 mm).

Salihović et al. (2021) [57] described the synthesis of two SBs deriving from l-cysteine, namely 2-((2-chlorobenzylidene)amino)-3-mercaptopropanoic acid (**22**) and 3-mercapto-2-((2-methoxybenzylidene)amino)propanoic acid (**23**), and an antimicrobial activity evaluation against five Gram-positive (*S. aureus, B. subtilis, Clostridium sporogenes, M. luteus* and *Microccocus flavus*), five Gram-negative (*E. coli, P. aeruginosa, Proteus hauseri, K. pneumoniae, Salmonella enterica* and subsp. *enterica* serovar Enteritidis) standard bacterial strains and some yeasts (*A. brasiliensis, S. cerevisiae* and *C. albicans*). Compound **22** showed higher antimicrobial activity against all tested bacteria (MIC = 1.284 mM), while compound **23** showed lower activity (MIC = 2.612 mM) compared to amikacin (MIC between 0.08 and 0.111 mM). The higher antifungal activity was found against *A. brasiliensis* for both compounds (MIC = 1.284 mM and 1.306 mM, respectively) versus amphotericin B (MIC = 0.044 mM).

Srinivasan et al. (2021) [58] described the synthesis and antibacterial evaluation of two pyrene-based SBs, 4-[(5-pyren-1-yl-thiophen-2-ylmethylene)-amino]-phenol (**24**) and 4-[(4-pyren-1-yl-benzylidene)-amino]-phenol (**25**), against two strains of *P. aeruginosa,* 9027 and 27853. The authors determined that **24** was more active as an antibacterial when compared to **25**; however, MIC or IZD values were not given. The treatment with compound **24** showed a minimum effect on the cell viability at a concentration range from 100 to 500 μg/mL. It was only found to be cytotoxic, i.e., almost 70% cell growth inhibition was evident, at a high concentration of **24**, i.e., 1000 μg/mL.

Sumrra et al. (2021) [59] reported a study on two mono-SBs, 4-[(5-amino-1*H*-1,2,4-triazol-3-yl)imino]methylbenzene-1,3-diol (**26**) and 2-[(5-amino-1*H*-1,2,4-triazol-3-yl)imino]methyl}-6-methoxyphenol (**27**), and one bis-SBs, 2,2′-{1*H*-1,2,4-triazole-3,5-diylbis [nitrilomethylylidene]}bis(6-methoxyphenol) (**28**), along with their metal complexes. An antibacterial activity evaluation was carried out for five bacterial strains (*Halomonas halophila, Chromohalobacter israelensis, E. coli, Chromohalobacter salexigens* and *Halomonas salina*). They exhibited antibacterial activity, and **26** was found to be more active against *C. salexigens* and *H. salina* and **27** was found to be more active against *E. coli* and *C. salexigens*, while **28** showed higher activity against *E. coli* and *H. salina.* Streptomycin, which was used as a reference, showed IZD values between 20 and 23 mm.

Ragi et al. (2021) [60] recently studied two SBs, 2,2′-(5,5-dimethylcyclohexane-1,3-diylidene)bis(azan-1-yl-1-ylidene)diphenol (**29**) and *N*,*N*′-(5,5-dimethylcyclohexane-1,3-diylidene)dianiline (**30**), against *S. aureus* and its target proteins after pre-filtering the drug-like properties of the compounds using the Lipinski rule of five. The compounds showed activity similar to that of ampicillin against *S. aureus* (IZD = 26 and 25 mm, respectively, versus 30 mm of ampicillin, all measured at 500 μg disc^−1^ concentration). The mechanisms by which these molecules can inhibit the growth of *S. aureus* were established by molecular docking studies on six different target proteins of *S. aureus*: sortase-A, clumping factor A (ClfA), dihydrofolate reductase (DHFR), DNA gyrase, undecaprenyl diphosphate synthase (UPPS) and dehydrosqualene synthase (CrtM) (PDB ID: 1T2P, 1N67, 2W9S, 3U2D, 4H8E and 2ZCO, respectively). Both SBs showed good binding affinity for the target protein DHFR, although in different sites. Thus, the authors suggested that the appreciable growth-inhibitory power exerted by compounds DmChDp and DmChDa against *S. aureus* could be related to the deactivation of dihydrofolate reductase [58].

Singhal et al. (2021) [61] studied 15 novel bis indole-based SBs as antibacterials. Comparative analyses against *S. aureus* and *E. coli* showed a higher inhibition of bis-SBs than the reference drug ciprofloxacin and their mono counterparts, the mono-SBs. The hexyl linker-based bis-SBs **31**–**33** depicted higher antibacterial action at 50 μg/mL, and the inhibitory effect increased with the increasing number of carbon atoms on the linker chain. Specifically, bis-SB **33** was the most active, showing MIC values of 37 and 34 mm against *E. coli* and *S. aureus*, respectively, compared to ciprofloxacin (IZD = 14 mm and 17 mm, respectively). Molecular docking studies on this compound were carried out with CT-DNA (PDBID:1BNA) and SARS-CoV-2 Mpro (3CL protease, PDBID:6LU7) via ultraviolet-visible and fluorescence spectroscopy techniques. Compound **33** proved its efficacy as a potential DNA binder and antiviral agent.

**Table 1:** Schiff bases with antibacterial activity.

Structure	Compd	Antimicrobial Activity	Ref
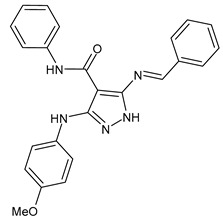	**1**	MIC = 7.81 µg/mL (*S. epidermidis*)	[45]
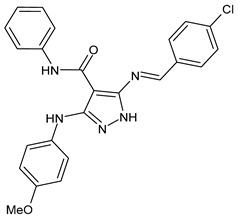	**2**	MIC = 7.81 µg/mL (*S. epidermidis*) MIC = 15.62 µg/mL (*A. baumanni*)	[45]
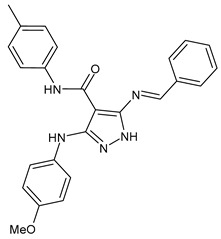	**3**	MIC = 7.81 µg/mL (*S. epidermidis*) MIC = 15.62 µg/mL (*A. baumanni*)	[45]
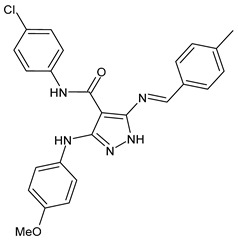	**4**	MIC = 7.81 µg/mL (*E. faecalis*)	[45]
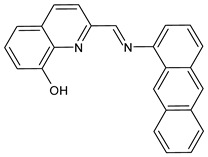	**5**	IZD = 13 mm (*B. cereus*)	[47]
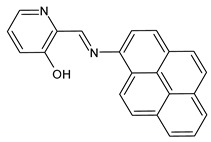	**6**	IZD = 16 mm (*B. cereus*) IZD = 14 mm (*E. coli* 10536)	[47]
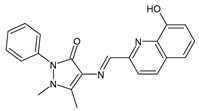	**7**	MIC = 25 µg/mL (*M. luteus* B1018) MIC = 12.5 µg/mL (*S. aureus* 23235)	[48]
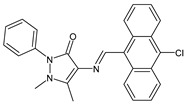	**8**	MIC = 25 µg/mL (*M. luteus* B1018) MIC = 12.5 µg/mL (*S. aureus* 23235) MIC = 12.5 µg/mL (*A. niger* 9642)	[48]
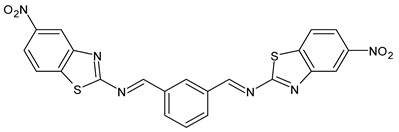	**9**	IZD = 14 mm (*S. aureus* MTCC 1144) IZD = 15 mm (*P. acnes* MTCC 1951)	[49]
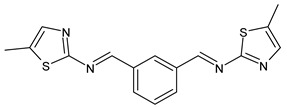	**10**	IZD = 10 mm (*S. aureus* MTCC 1144) IZD = 14 mm (*P. acnes* MTCC 1951)	[49]
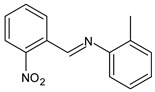	**11**	MIC = 250 µg/mL (*E. coli*) MIC = 125 µg/mL (*S. thyphymurium*)	[50]
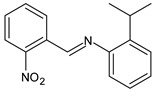	**12**	MIC = 125 µg/mL (*E. coli*) MIC = 15.625 µg/mL (*S. thyphymurium*) MIC = 7.81 µg/mL (*P. aeruginosa*)	[50]
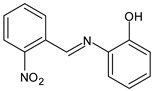	**13**	MIC = 62.50 µg/mL (*E. coli*) MIC = 62.50 µg/mL (*S. thyphymurium*)	[50]
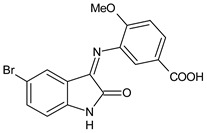	**14**	MIC = 625 μg/mL (*S. aureus* 25923) MIC = 78 μg/mL (*P. aeruginosa* clinical strain) IZD = 15 mm (*P. aeruginosa* clinical strain)	[51]
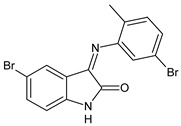	**15**	MIC = 625 μg/mL (*K. pneumoniae* 700603)	[51]
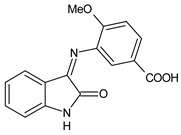	**16**	MIC = 78 μg/mL (*P. aeruginosa* clinical strain) IZD = 24 mm (*P. aeruginosa* clinical strain)	[51]
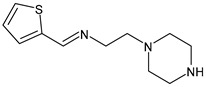	**17**	IZD = 12 mm (*S. aureus* 25923) IZD = 11 mm (*S. aureus* MRSA) IZD = 9 mm (*P. aeruginosa* 27853)	[52]
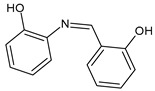	**18**	IZD = 32 mm (*S. epidermidis*) IZD = 32 mm (*P. aeruginosa*)	[53]
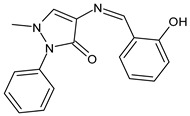	**19**	IZD = 32.5 mm (*S. aureus*)	[53]
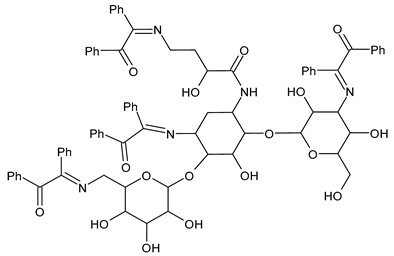	**20**	IZD = 37 mm (*S. aureus*) IZD = 37 mm (*E. coli*)	[54]
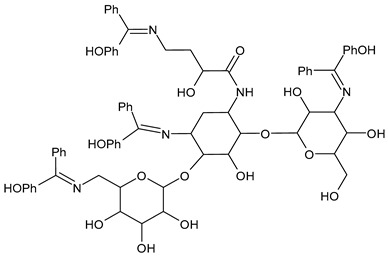	**21**	IZD = 321 mm (*S. aureus*) IZD = 38 mm (*E. coli*)	[54]
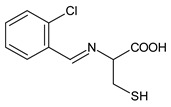	**22**	MIC = 1.284 mM (*S. aureus* 6538; *B. subtilis* 6633; *C. sporogenes* 19404; *M. luteus* 4698; *M. flavus* 10240) MIC = 1.284 mM (*E. coli* 25922; *S. aeruginosa* 9027; *Proteus hauseri* 13315; *K. Pneumoniae* 10031; *Salmonella enterica* subsp. *enterica* serovar Enteritidis 13076) MIC = 1.284 mM (*A. brasiliensis* 16,404)	[55]
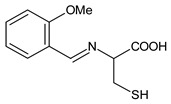	**23**	MIC = 2.612 mM (*S. aureus* 6538; *B. subtilis* 6633; *C. sporogenes* 19404; *M. luteus* 4698; *M. flavus* 10240) MIC = 2.612 mM (*E. coli* 25922; *S. aeruginosa* 9027; *Proteus hauseri* 13315; *K. Pneumoniae* 10031; *Salmonella enterica* subsp. *enterica* serovar Enteritidis 13076) MIC = 1.306 mM (*A. brasiliensis* 16404)	[55]
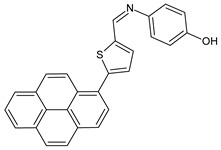	**24**	MIC and/or IZD not given	[56]
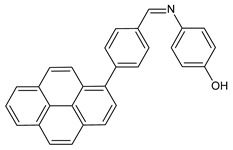	**25**	MIC and/or IZD not given	[56]
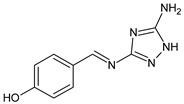	**26**	IZD = 12 mm (*C. salexigens* and *H. salina*) IZD = 10 mm (*H. halophila*)	[57]
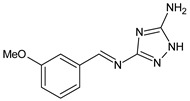	**27**	IZD = 14 mm (*C. salexigens*) IZD = 12 mm (*E. coli*) IZD = 10 mm (*C. israelensis*)	[57]
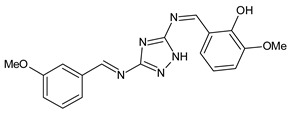	**28**	IZD = 14 mm (*C. salexigens*) IZD = 13 mm (*E. coli*)	[57]
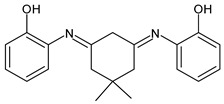	**29**	IZD = 26 mm (at 500 μ/disc)	[58]
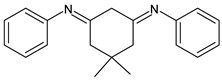	**30**	IZD = 25 mm (at 500 μg/disc)	[58]
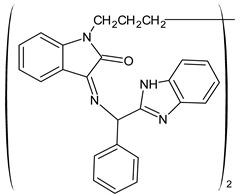	**31**	IZD = 32 mm (*E. coli*) IZD = 22 mm (*S. aureus*)	[59]
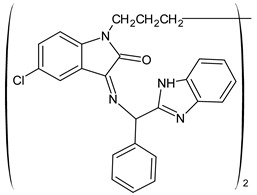	**32**	IZD = 34 mm (*E. coli*) IZD = 28 mm (*S. aureus*)	[59]
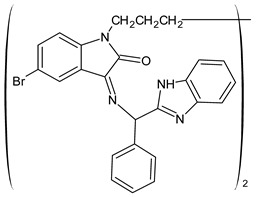	**33**	IZD = 37 mm (*E. coli*) IZD = 34 mm (*S. aureus*)	[59]

**Figure 2:**
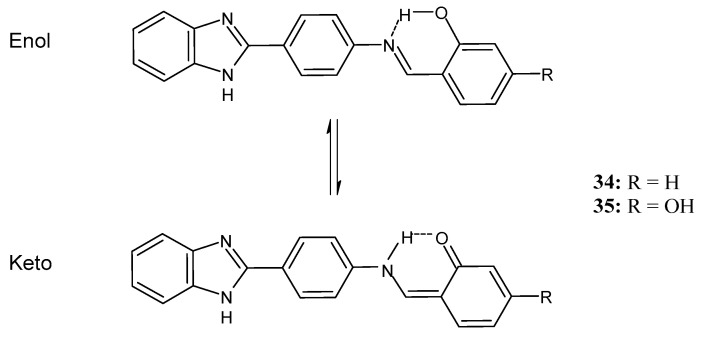
Keto–enol equilibrium of **34** and **35** SBs.

A recent study by Aragón-Muriel et al. (2021) [62] investigated the antibacterial potential of four benzimidazole-based Schiff bases and their metal complexes against two Gram-positive strains (*S. aureus* 25923, *Listeria monocytogenes* 19115) and two Gram-negative strains (*E. coli* 25922, *P. aeruginosa* 27583). As generally observed, the activity of the complexes was higher than that of SBs alone. Moreover, isomerism in SBs seemed to play a crucial role, as is often the case for compounds with biological activity [63]. The major activity found in the study by Aragón-Muriel et al. (2021) [62] was shown by **34** and **35** (MIC = 250 ng/mL against *S. aureus* versus 0.5 ng/mL of ciprofloxacin). Despite the low activity of SBs, this study provided novel insights into the mechanisms through which the compound exerts antibacterial action, suggesting that interactions with the bacterial membrane or interactions with DNA could be involved. The types of interactions that occurred between nucleic acid and new compounds was studied by UV/V spectrophotometry. Differences can be found in the tautomers deriving from the keto–enol equilibrium (Figure 2). The interaction of DNA with these systems would result in the stabilization of one of the tautomers when a DNA–compound complex is formed. The interaction with DNA seemed to favor the keto form of **34**, while the union of **35** with DNA strands stabilized the enol form of **35**.

### 2.2. Schiff Bases with Antifungal Activity

All the data described in this paragraph are summarized in Table 2. Some studies did not report MICs or IZDs but did provide inhibitory indexes (IIs).

Magalhães et al. (2020) [64] described the synthesis and the in vitro antifungal evaluation of 23 cinnamyl SBs. Six of them showed antifungal activities against strains of *Candida*, *Aspergillus*, *Fonsecaea* and, specifically, *Cryptococcus* species. Compounds **36** and **37** showed MIC values more than two-fold lower than that of the reference fluconazole against all the *Cryptococcus neoformans* strains (MIC = 1.33 µg/mL and 1.4 µg/mL, respectively, versus fluconazole, 5.2 µg/mL) and *Cryptococcus gattii* strains (MIC = 5.3 µg/mL and 2.8 µg/mL, respectively, versus fluconazole, 9.2 µg/mL), while cinnamyl SB **38** was as potent as fluconazole against all strains from both *Cryptococcus* species. No significant cytotoxic effects were observed for SBs against human lung (MRC-5, human fetal lung fibroblast), kidney (HEK 293, human embryonic kidney) or red blood cells, all presenting selective indexes higher than 10.

Chen et al. (2020) [65] described a structural modification of inulin by SBs in order to improve its biological activity. The antifungal studies against three kinds of plant pathogenic fungi (*Botrytis cinerea, Fusarium oxysporum* f. sp. cucumerium Owen and *Phomopsis asparagi*), according to Guo’s method [66], showed that synthetic inulin derivatives demonstrated a broad antifungal spectrum. At 1.6 mg/mL, the IIs of **39** were 93%, 83% and 82% against *B. cinerea, F. oxysporum* f. sp. *cucumerium* Owen and *P. asparagi*, respectively. The lipophilic characteristics of **39** due to the presence of the benzene ring and the acetyl groups allow this compound to easily penetrate into the cell, leading to apoptosis. Moreover, the new inulin derivatives modified with the Schiff bases showed an excellent metal binding property, suppressing the growth of microbes by their interaction with cellular components.

A recent study by Hamad et al. [67] described the synthesis of SBs of sulphonamides and their antifungal activity against multidrug-resistant *Candida auris*, an emerging fungal pathogen with a high mortality rate, which is difficult to identify and carries considerable risks of healthcare outbreaks [68]. In 2020, diverse cases of candidemia outbreaks related to COVID-19 in intensive care units in hospitals and severe fungal coinfections have been reported worldwide [69]. The SBs of sulphonamides were first tested against a multispecies panel of *Candida* strains (*C. auris* TDG1912*, Candida albicans* NCPF3281 *and* NCPF3179, *Candida glabrata* NCPF8018, *Candida krusei* NCPF3876, *Candida tropicalis* NCPF8760 and *Candida parapsilosis* NCPF3209). Compound **40** showed interesting MIC values against almost all the strains in the range of 4–32 µg/mL (versus 0.5–>128 µg/mL of fluconazole). Thus, it was tested against a larger panel of multidrug-resistant *C. auris* strains (TDG2506, TDG2512, TDG1102, TDG2211, NCPF8984, NCPF8977 and NCPF8971), and its antifungal activity was confirmed with MICs of 4–16 µg/mL, which are very low considering that in four cases (TDG2506, TDG1102, TDG2211 and NCPF8984) the MIC value for fluconazole was ≥128 µg/mL. Compound **41** showed the same results of **40** only on the extended panel [64].

Bhagwatrao Biradar et al. (2021) [70] reported the synthesis of SBs derived from dehydroacetic acid (DHA) and evaluated their antimicrobial activity. The most interesting compounds were **42,** showing an IZD = 19 mm against *A. niger* (griseofulvin IZD = 22 mm) and **43** against *C. albicans* (IZD = 21 mm versus griseofulvin, IZD = 25 mm).

**Table 2:** Schiff bases with antifungal activity.

Structure	Compd	Antimicrobial Activity	Ref
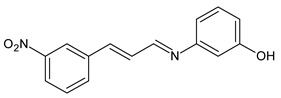	**36**	MIC = 1.33 µg/mL (*C. neoformans*) MIC = 5.3 µg/mL (*C. gatii*)	[64]
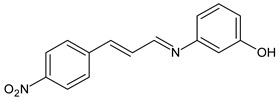	**37**	MIC = 1.4 µg/mL (*C. neoformans*) MIC = 2.8 µg/mL (*C. gatii*)	[64]
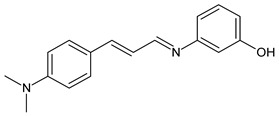	**38**	MIC = 3.2 µg/mL (*C. neoformans*) MIC = 8.0 µg/mL (*C. gatii*)	[64]
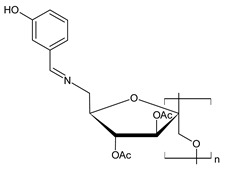	**39**	*II =* 93% (*B. cinerea*) *II =* 83% (*F. oxysporum* f. sp. *cucumerium* Owen), *II =* 82% (*P. asparagi*)	[65]
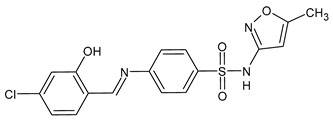	**40**	MIC = 16 µg/mL (*C. auris* TDG1912; *C. albicans* NCPF3281) MIC = 16–32 µg/mL (*C. albicans* NCPF3179) MIC = 4–8 µg/mL (*C. glabrata* NCPF8018) MIC = 8–16 µg/mL (*C. auris* NCPF8971) MIC = 16 µg/mL (*C. auris* TDG2512, TDG2506, NCPF8984, NCPF8977) MIC = 16–32 µg/mL (*C. auris* TDG1102, TDG2211)	[67]
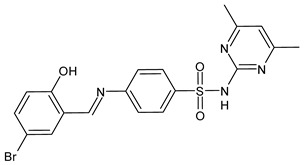	**41**	MIC = 8–16 µg/mL (*C. auris* NCPF8971) MIC = 16 µg/mL (*C. auris* TDG2512, TDG2506, NCPF8984, NCPF8977) MIC = 16–32 µg/mL (*C. auris* TDG1102, TDG2211)	[67]
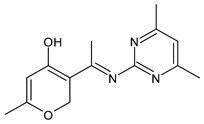	**42**	IZD = 19 mm (*A. niger*)	[70]
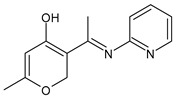	**43**	IZD = 21 mm (*C. albicans*)	[70]

## 3. Chitosan-Based Schiff Bases (CBSs)

Chitosan (*N*-deacetylated product of chitin) is a natural biopolymer composed of β-d-glucosamine and *N*-acetyl-β-d-glucosamine units with a 1,4-linkage. Chitosan is an attractive material because of its properties such as immunological activity, biocompatibility, low toxicity and biodegradability. Chitosan is insoluble in water and most organic solvents, which seriously limits its application. CBSs result from the chemical modification of chitosan via imine functionalization (RR′C=N-R″; R = alkyl/aryl, R′ = H/alkyl/aryl and R″ = chitosan ring). They are usually synthesized by the Schiff condensation reaction between chitosan’s amino groups and carbonyl compounds with the removal of water molecules. The antibacterial applications of chitosan derivatives are numerous [71,72]. Recently, two chitosan Schiff bases were used for the removal of Cr(VI) from wastewater with high efficiency [73,74]. Antimicrobial studies of CBSs are reported in Table 3.

Barbosa et al. (2017) [75] described several biopolymeric Schiff bases of chitosan and different salicylaldehydes and their complexes with palladium(II) and platinum(II). They were tested for antimicrobial activity in vitro against two common bacterial and fungal plant pathogens, *Pseudomonas syringae* pv. *tomato* and *Fusarium graminearum*, respectively, and for their antiproliferative activity against human MCF-7 breast cancer cells. Compared to the unmodified chitosan, CBSs and their complexes had higher antibacterial effects against *P. syringae* but were highly toxic against MCF-7 cells. In particular, the MIC value of **44** was 25 µg/mL (versus >50 µg/mL of purified chitosan). The antifungal effects against *F. graminearum* were less pronounced compared to the nonmodified chitosan (**44**, MIC = 50 µg/mL versus 30 µg/mL of purified chitosan), suggesting diverse modes of action for the two fungal species.

Wei et al. (2019) [76] synthesized eight CBSs and quaternary ammonium salts in order to improve the antioxidant and antifungal activity of chitosan. The antifungal activity against *F. oxysporum* f. sp. *cucumerium*, *B. cinerea* and *F. oxysporum* f. sp. *niveum* was evaluated using a mycelium growth rate test. CBSs exhibited enhanced antifungal activity when compared to chitosan, especially at 1.0 mg/mL. Chitosan II was 16.9% at 1.0 mg/mL. The most interesting were 6-[4-(2,3-dihydroxyl-benzimide) pyridine] acetyl-2-*N*,*N*,*N*-trimethyl-chitosan chloride (**45**) and 6-[4-(2,3,4-trihydroxyl-benzimide) pyridine] acetyl-2-*N*,*N*,*N*-trimethyl-chitosan chloride (**46**), which showed IIs >90.0% at 1.0 mg/mL against *F. oxysporum* f. sp. *cucumerium* and *B. cinerea*. The chitosan derivatives also showed stronger antioxidant activity than chitosan. It was suggested that the higher density of the positive charge contributes to the antifungal activity. The positive charge could interact with the anionic substances, such as glucan, mannan, proteins and lipids to form a lipophilic layer around the cell, which could prevent nutrient exchange in the cells. The authors posited that the higher antifungal activity of **45** and **46** might be due to the presence of phenolic groups.

Hassan et al. (2018) [77] described the ability of two antimicrobial chitosan SBs, **47** and **48**, to boost the antimicrobial activity of native chitosan against Gram-positive bacteria (*S. aureus* and *B. cereus*), Gram-negative bacteria (*E. coli*, *P. aeruginosa* and *Salmonella* sp.) and *C. albicans* fungal species. The antimicrobial activity of SB **47** was significantly higher than that of SB **48** and chitosan (IZD = 11.5, 12.6, 13, 13.9, 13.6 and 11.4 mm (against *E. coli*, *P. aeruginosa*, *Salmonella* spp. and *S. aureus*, *C. albicans*) using erythromycin (IZD = 11 and 12.6 mm against *E. coli* and *Salmonella* spp.) and nystatine (IZD = 15.2 mm against *C. albicans*) as reference drugs. The highest concentration of compound **47** could inhibit the growth of Gram-positive bacteria up to 99%, whereas compound **48** recorded the maximum inhibition rate against Gram-positive bacteria, by approximately 82%. The higher action of compound **47** on Gram-positive bacteria than Gram-negative bacteria, which appeared at 250 μg/mL, was attributed to the difference of the cell wall structures. The cytotoxicity of the developed materials was estimated by MTT assay in fibroblast cells. The cellular toxicity of SBs I and II at 200 mg were 5.2% and 6.3%, respectively, thus substantiating their safety.

Ali et al. (2018) [78] studied a chitosan derivative, a methyl acrylate chitosan-bearing *p*-nitrobenzaldehyde (**49**) Schiff base, and examined its antibacterial activity against MDR Gram-positive bacteria MDR *S. aureus* (MDR-SA) and MDR Gram-negative bacteria MDR *P. aeruginosa* (MDR-PA), MDR *K. pneumoniae* (MDR-KP) and MDR *E. coli* (MDR-EC) by using the agar-well diffusion method. The chitosan derivative **49** showed significantly higher antibacterial activity against MDR-SA and MDR-PA compared to other bacteria. MDR *S. aureus* 4 (MDR-SA-04) and MDR *P. aeruginosa* 9 (MDR-PA-09) strains were found to be more susceptible Gram-positive and Gram-negative bacterial strains to **49**, respectively. The highest mean IZDs ranged from 15.0 ± 0.23 to 28.5 ± 0.03 mm and from 10.5 ± 0.03 to 22.1 ± 0.05 mm, respectively. On the other hand, **49** revealed the highest antibacterial activity, showing an MIC value of 6.25 μg/mL for both bacterial strains. Moreover, it exhibited antibiofilm activity against MDR-PA-09, as assessed by using a microtiter plate. The compound also showed antioxidant and anti-inflammatory activities, as determined by the 2,2-diphenyl-2-picrylhydrazyl (DPPH) method and by albumin denaturation, membrane stabilization of red blood cells assay and proteinase inhibition methods.

Recently, Wei et al. (2021) [79] reported another work regarding the bis-Schiff bases of chitosan derivatives tested for their antifungal action against three plant pathogenic fungi (*B. cinerea*, *Fusarium oxysporum* f. sp. *cucumerinum* and *Fusarium oxysporum* f. sp. *Niveum*). The results show that the bis-Schiff bases of chitosan showed higher antifungal activity than chitosan, especially at 1.0 mg/mL. Specifically, derivatives bearing halogeno-benzenes (**50**–**52**) showed IIs higher than 95% at 1.0 mg/mL against *B. cinerea* (versus chitosan 23.8%), and this high activity was attributed to the halogen with stronger electron-withdrawing properties.

## 4. Nanoformulations and Nanomedicines

Nanotechnology has recently emerged as a very interesting field of research with countless biomedical science applications. It is described as the design, characterization and application of structures, devices and systems by monitoring shape and size at a nanometer scale (1 nm to 100 nm) [80]. Silver, gold and copper nanoparticles have been widely described for their anticancer properties [81], and silver and gold nanoparticles have also shown antibacterial and antiviral activities [82,83]. In addition, experimental evidence regarding nanoparticles with Schiff bases for the diagnosis and treatment of tumors have been also reported. Some prodrug nanomedicines deriving from Schiff bases with doxorubicin and polyethylene glycol (PEG), one of the most widely used hydrophilic polymer approved by Food and Drug Administration (FDA) with negligible toxicity, have been described for their antitumor activity [84]. Moreover, the functionalization of chitosan nanoparticles with the anticancer 5-fluorouracil to obtain chitosan nanocarriers through Schiff base formation has been studied [85].

### 4.1. Nanoparticles of Schiff Bases

Several studies on the ability of nanoparticles to enhance antibacterial activities have also been reported. Several nanocojugates, i.e., Schiff base complexes with zinc conjugated to silver nanoparticles, showed excellent antimicrobial activity against *S. aureus* [86]. A recent work by Minhaz et al. (2020) [87] compared the activity of an SB (**53**) and silver nanoparticles of this compound (**53-AgNPs**), studied as nanoprobes. The latter exhibited significant antibacterial and anticancer activities and was also used as sensitive protocol for the detection and quantification of heavy metal Hg(II) in tap water. Regarding antibacterial activity, the IZDs values for **53** were 8.0, 9.0, 7.0 and 7.0 mm against *S. aureus*, *B. subtilis*, *P. aeruginosa* and *E. coli*, respectively, and **53-AgNPs** achieved IZD values of 18.0, 18.0, 10.0 and 10.0 mm, respectively (values for vancomycin were 18.0, 25.0, 12.0 and 13.0 mm, respectively). Cytotoxicity results show that **53-AgNPs** at a concentration of 100 mg/mL induced DU-145 cell toxicity and caused 94% cell death after treatment up to 72 h. Moreover, in normal cells (L-929) death rate was lower (up to 1.5%) as compared to cancer cells, and this was indicative of a selective action of this compound.

### 4.2. Nanoparticles of Schiff Bases with Chitosan

Biomaterials based on chitosan modified with nanoparticles have shown promising results as antibacterials [88,89,90]. In fact, chitosan-based nanoparticles have several advantages including efficiency, cost-effectiveness, biocompatibility, biodegradability, non-toxicity and non-immunogenicity [91].

Abdel-Monem et al. (2020) [92] described the synthesis of nanocomposites of salicylaldehyde and anisaldehyde carboxymethyl chitosan–Schiff bases (**54** and **55**, respectively, Table 4) also loaded with silver nanoparticles (**54-AgNO_3_** and **55-AgNO_3_**) and studied their antibacterial activities against Gram-positive bacteria (*B. subtilis*, *S. aureus* and *Streptococcus faecalis*) and Gram-negative bacteria (*E. coli*, *Neisseria gonorrhoeae* and *P. aeruginosa*). CMCs derivatives presented remarkable antimicrobial activity, which was increased using silver (AgNO_3_ 5%). Ampicillin was used as a reference (IZD = 20, 18, 18, 22, 20 and 17 mm against *B. subtilis*, *S. aureus*, *S. faecalis*, *E. coli*, *N. gonorrhoeae* and *P. aeruginosa*, respectively). SB **54** was more active than SB **55**. Moreover, **54-AgNO_3_** was more active than **55-AgNO_3_**. Interestingly, compound **54-AgNO_3_** was even more active against *P. aeruginosa* than the reference (ampicillin; IZD = 17 mm). Cytotoxic activity was evaluated against liver HePG2 and breast MCF7 cancer cells. CMC-SBs showed lower cytotoxicity than carboxymethyl chitosan against both tumor cell lines. The cytotoxicity was even lower when considering CMC-SBs silver nanoparticles.

Recently, a CSB (**56**) and its CSB-NiFe nanocomposite (**56-NiFe**) were tested as antimicrobials against two Gram-positive (*S. aureus* and *B. cereus*) and two Gram-negative (*E. coli* and *P. aeruginosa*) bacteria by the agar-well diffusion method [93]. The results obtained show that the antibacterial activities of the compounds **56** and **56-NiFe** were stronger than that of pure chitosan. The authors postulated that the compounds show weak antibacterial activity against *P. aeruginosa*, medium activity against *B. cereus* and good activity against *S. aureus* and *E. coli*. However, the MIC and/or IZD values are not given.

## 5. Summary

SBs are inexpensive compounds and easy to synthesize. They display structural and electronic features that enable their application in a large number of research fields, such as analytical, inorganic and organic chemistry. Moreover, their antibacterial, antifungal, anticancer, urease inhibitor and antioxidant effects have been widely reported. Additionally, SBs have shown antiglycation activities, anti-inflammatory, antitumor, antiviral, antipyretic and anti-HIV-1 abilities. Nowadays, the need for more effective antibacterial and antifungal therapies is pressing due to the high mortality rates that are associated with bacterial and fungal diseases as well as the growing number of multidrug-resistant strains. This review summarizes the antimicrobial effects of SBs as described in the recent literature and highlights the importance of CSBs. Chitosan is a product of the deacetylation of chitin, which is widely found in nature. It has proved antibacterial activity, good biodegradation, outstanding biocompatibility, is non-toxic and has excellent chemical and physical properties. As a result, chitosan and its derivatives have been widely tested in the antimicrobial field, showing antibacterial activity against Gram-positive and Gram-negative bacteria as well as antifungal potential. Interestingly, chitosan nanoparticles exhibit antibacterial activity. The most recent studies on CBSs and their nanoparticles as antibacterials have been herein summarized. It is worth mentioning that antibacterial and antitumor activities are generally higher when organic compounds, including SBs, are complexed with metals. Hence, the antimicrobial action of SBs reviewed herein may be taken into account to favor the development of novel metal complexes with improved antimicrobial capacities. Finally, the antibacterial activity of SBs, along with their known anticorrosive potential, might also represent great potential for their future application in several types of surgeries.

## Figures and Tables

**Table 3:** Structure and antibacterial activity of chitosan-based Schiff bases.

Structure	Compd	Antimicrobial Activity	Ref
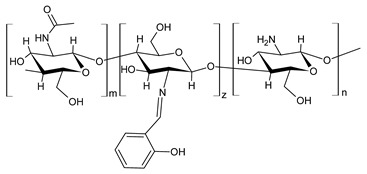	**44**	MIC = 25 µg/mL (*P. syringae*) MIC = 50 µg/mL (*F. graminearum*)	[75]
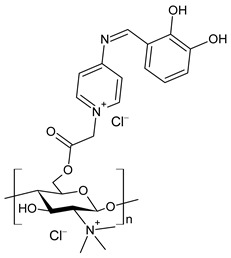	**45**	II > 90%	[76]
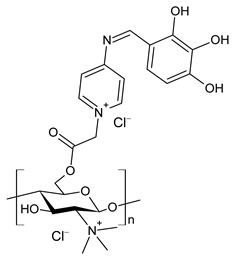	**46**	II > 90%	[76]
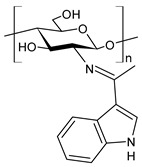	**47**	IZD = 17.7 mm (*E. coli*) IZD = 17.2 mm (*P. aeruginosa*) IZD = 17.1 mm (*Salmonella* spp.) IZD = 18.9 mm (*S. aureus*) IZD = 18.1 mm (*C. albicans*)	[77]
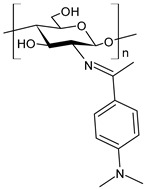	**48**	IZD = 13.7 mm (*E. coli*) IZD = 14.4 mm (*P. aeruginosa*) IZD = 14.7 mm (*Salmonella* spp.) IZD = 15.1 mm (*S. aureus*) IZD = 15.5 mm (*C. albicans*)	[77]
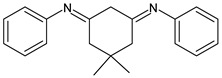	**49**	IZD = 28.5 mm (MDR-SA-04) IZD = 22.1 mm (MDR-PA-09)	[78]
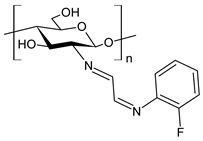	**50**	II = 96.7% (*B. cinerea* at 1.0 mg/mL)	[79]
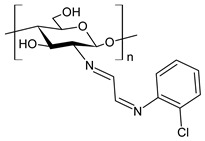	**51**	II = 96.0% (*B. cinerea* at 1.0 mg/mL)	[79]
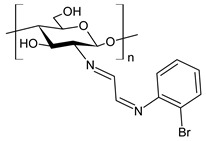	**52**	II = 95.8% (*B. cinerea* at 1.0 mg/mL)	[79]

**Table 4:** Structure and antibacterial activity of nanoparticles of SBs and CBSs.

Structure	SB or SB/Nanoparticle	Antimicrobial Activity	Ref
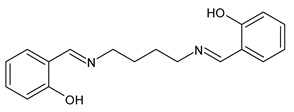	**53**	IZD = 8.0 mm (*S. aureus*) IZD = 9.0 mm (*B. subtilis*) IZD = 7.0 mm (*P. aeruginosa*) IZD = 7.0 mm (*E. coli*)	[87]
	**53-AgNPs**	IZD = 18.0 mm (*S. aureus*) IZD = 18.0 mm (*B. subtilis*) IZD = 10.0 mm (*P. aeruginosa*) IZD = 10.0 mm (*E. coli*)	[87]
**Nanoparticles of Schiff bases with chitosan**			
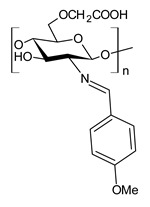	**54**	IZD = 10 mm (*B. subtilis*) IZD = 11 mm (*S. aureus*) IZD = 10 mm (*S. faecalis*) IZD = 12 mm (*E. coli*) IZD = 13 mm (*N. gonorrhoeae*) IZD = 13 mm (*P. aeruginosa*)	[92]
	**54-AgNO_3_**	IZD = 15 mm (*B. subtilis*) IZD = 15 mm (*S. aureus*) IZD = 14 mm (*S. faecalis*) IZD = 21 mm (*E. coli*) IZD = 19 mm (*N. gonorrhoeae*) IZD = 20 mm (*P. aeruginosa*)	[92]
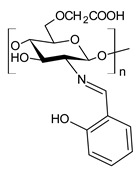	**55**	IZD = 9 mm (*B. subtilis*) IZD = 10 mm (*S. aureus*) IZD = 10 mm (*S. faecalis*) IZD = 11 mm (*E. coli*) IZD = 12 mm (*N. gonorrhoeae*) IZD = 12 mm (*P. aeruginosa*)	[92]
	**55-AgNO_3_**	IZD = 13 mm (*B. subtilis*) IZD = 13 mm (*S. aureus*) IZD = 14 mm (*S. faecalis*) IZD = 15 mm (*E. coli*) IZD = 14 mm (*N. gonorrhoeae*) IZD = 16 mm (*P. aeruginosa*)	[92]
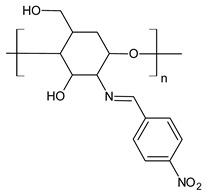	**56**	MIC and/or IZD not given. Only photos are shown	[93]
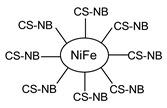	**56-NiFe**	MIC and/or IZD not given. Only photos are shown	[93]

## Data Availability

Not applicable.

## Abbreviations

*A. baumanni*	*Acinetobacter baumanni*
ADMET	Absorption, distribution, metabolism, excretion and toxicity
*A. niger*	*Aspergillus niger*
*B. cereus*	*Bacillus cereus*
*B. cinerea*	*Botrytis cinerea*
*B. subtilis*	*Bacillus subtilis*
*C. albicanss*	*Candida albicans*
*C. auris*	*Candida auris*
*C. glabrata*	*Candida glabrata*
CBSs	Chitosan-based Schiff bases
ClfA	Clumping factor A
CrtM	Dehydrosqualene synthase
DFT	Density functional theory
DHFR	Dihydrofolate reductase
*E. coli*	*Escherichia coli*
*E. faecalis*	*Enterococcus faecalis*
FDA	Food and Drug Administration
*F. graminearum*	*Fusarium graminearum*
*F. oxysporum*	*Fusarium oxysporum*
IC_50_	Concentration which kills or inhibits cell viability by 50%
II	Inhibitory Index
IZD	Inhibition zone diameter
*K. pneumoniae*	*Klebsiella pneumoniae*
MEPS	Molecular electrostatic potential surface
*M. luteus*	*Micrococcus luteus*
MTCC	Microbial Type Culture Collection
*N. gonorrhoeae*	*Neisseria gonorrhoeae*
*P. acnes*	*Propionic bacteria acnes*
*P. aeruginosa*	*Pseudomonas aeruginosa*
*P. asparagi*	*Phomopsis asparagi*
*P. syringae*	*Pseudomonas syringae*
PEG	Polyethylene glycol
QSAR	Quantitative structure activity relationship
SBs	Schiff bases
*S. aureus*	*Staphylococcus aureus*
*S. epidermidis*	*Staphylococcus epidermidis*
*S. faecalis*	*Streptococcus faecalis*
UPPS	Undecaprenyl diphosphate synthase
